# Clinical Utility of Artificial Intelligence Algorithms to Enhance Wide-Field Optical Coherence Tomography Angiography Images

**DOI:** 10.3390/jimaging7020032

**Published:** 2021-02-10

**Authors:** Orlaith Mc Grath, Mohammad W. Sarfraz, Abha Gupta, Yan Yang, Tariq Aslam

**Affiliations:** Manchester Royal Eye Hospital, Central Manchester University Hospitals NHS Foundation Trust, Oxford Road, Manchester M13 9WL, UK; waseem.sarfraz@doctors.org.uk (M.W.S.); abha.gupta@mft.nhs.uk (A.G.); yan.yang-10@postgrad.manchester.ac.uk (Y.Y.); tariq.aslam@mft.nhs.uk (T.A.)

**Keywords:** optical coherence tomography, angiography, deep learning, denoise

## Abstract

The aim of this paper is to investigate the clinical utility of the application of deep learning denoise algorithms on standard wide-field Optical Coherence Tomography Angiography (OCT-A) images. This was a retrospective case-series assessing forty-nine 10 × 10 mm OCT-A1 macula scans of 49 consecutive patients attending a medical retina clinic over a 6-month period. Thirty-seven patients had pathology; 13 had none. Retinal vascular layers were categorised into superficial or deep capillary plexus. For each category, the retinal experts compared the original standard image with the same image that had intelligent denoise applied. When analysing the Superficial Capillary Plexus (SCP), the denoised image was selected as “best for clinical assessment” in 98% of comparisons. No difference was established in the remaining 2%. On evaluating the Deep Capillary Plexus (DCP), the denoised image was preferred in 35% of comparisons. No difference was found in 65%. There was no evidence of new artefactual features nor loss of anatomical detail in denoised compared to the standard images. The wide-field denoise feature of the Canon Xephilio OCT-A1 produced scans that were clinically preferable over their original OCT-A images, especially for SCP assessment, without evidence for causing a new artefactual error.

## 1. Introduction

Optical coherence tomography angiography (OCT-A) has recently emerged as a powerful non-invasive technique for imaging the microvasculature of the retina. The first clinical studies using this technology were published in 2014 [1], and since then, the technology has improved to allow even greater insight into retinal vascular anatomy [2]. OCT-A technology uses laser light reflectance of the surface of moving red blood cells to accurately depict blood vessels through different segmented areas of the eye. It visualizes the retina in a depth-resolved fashion, allowing the separate study of the retinal superficial and deep vascular plexuses [3].

Initially, the scanning range of OCT-A was limited to the macula [4]. However, imaging technology has evolved to allow scanning of larger areas of the posterior pole [5]. The Canon Xephilio OCT-A1 wide-field fundal view enables us to image 10 × 10 mm (and wider) segments of the retinal vasculature. This wide view allows improved visualization of conditions that can affect the more peripheral retina such as diabetic retinopathy [6], central serous chorioretinopathy [7], and vascular occlusion [8]. OCT-A has been shown to have higher contrast and resolution than fluorescein angiography (FFA), the gold standard for decades in evaluating retinal vasculature [3,9]. As OCT-A can be faster and safer due to its non-invasive nature, it has the potential to become implemented in clinical practice for many indications above dye-based angiography [10,11]. However promising, the technology still has limitations. For example, signal noise as well as projection artefact may complicate accurate interpretation of OCT-A images even in normal populations [12].

Studies have shown that averaging multiple en face OCT-A images improves image quality [13]. However, it takes much longer to obtain multiple OCT-A images in order to permit the averaging process than it does to take a single OCT-A image and is not always feasible in practice.

The novel Canon Xephilio OCT-A1 device uses in-built processing software that allows for a deep-learning-based algorithm for noise reduction (denoise) to reduce image noise, increase detail and improve visibility of vasculature in OCT-A images. This system for enhancing imaging involves a Convolutional Neural Net (CNN) that was trained on averaged OCT-A images to reduce noise (see Appendix A for a schematic of the deep learning denoise model used). A recent study by Kadomoto S. et al. [14] has demonstrated this denoising via deep learning as a faster alternative to averaging for OCT-A processing. It was shown to produce improved quality retinal microvasculature images compared to standard images in terms of objective outcome measures such as contrast-to-noise ratio (CNR) and peak signal-to-noise ratio (PSNR). The same research team also suggested that some artefactual errors were created by the denoise algorithms by comparing the original and denoised images to averaged images of the same patient as a gold standard. However, averaged images are also subject to imperfections: long visits can compromise image quality leading to loss of image detail, and excessive processing may lead to excessive smoothing of images [15]. Thus, comparison with averaged images may not necessarily provide unquestionable evidence of artefact.

For this study, we make a pragmatic assessment of the practical utility of intelligent denoise software on wide-field OCT-A1 images captured in a clinical setting. We investigate its utility as judged by retinal physicians comparing the two images side by side to determine which is clinically preferable. The retinal experts judged if there are any definite areas of the processed image where artefactual errors are evident but not present in the unprocessed image, according to their clinical experience. We study the deeper and superficial vessel layers as well as a wide field of view (10 × 10 mm), which have never previously been reported on. We will assess evidence for clinicians to use the denoised images in preference to standard images and whether they can safely be used without reverting to also scrutinizing the original images each time.

## 2. Materials and Methods

This was a retrospective, qualitative, cross-sectional case series study. The research was prospectively approved to study anonymised patient images by the Central Manchester University Foundation Trust Research and Development Office. This project was conducted in accordance with the study protocol and the ethical principles outlined by Good Clinical Practice (GCP) and the Declaration of Helsinki.

### 2.1. Participants

This was a study of anonymised OCT-A scans of one eye of 50 consecutive patients seen at Manchester Royal Eye Hospital Medical Retina Out-Patient Clinic, Manchester, UK, between September 2019 and March 2020. Patients attending the general medical retina clinic over this 7 month period were included in the study if they were over 4 years of age and if they had a Canon OCT-A1 scan captured as part of their standard of care. All patients underwent a comprehensive ophthalmic examination including, but not limited to, measurement of best-corrected visual acuity, slit lamp examination, and OCT-A imaging. The exclusion criteria for this study were OCT-A images of poor quality that hindered image analysis including significant media opacity, significant segmentation errors, and motion artefacts such as motion lines. The Canon image quality rating had to be higher than a score of 4 out of 10 for the scan to be included in the dataset. Eyes with high myopia (greater than minus 6 dioptres) or high astigmatism (more extreme than 3 dioptres) were excluded. The eye with the most clinical pathology was included in the study, but if no pathology was present or if pathology was bilateral, the left eye was selected for analysis.

### 2.2. OCT-A Image Capturing

**Figure 1:**
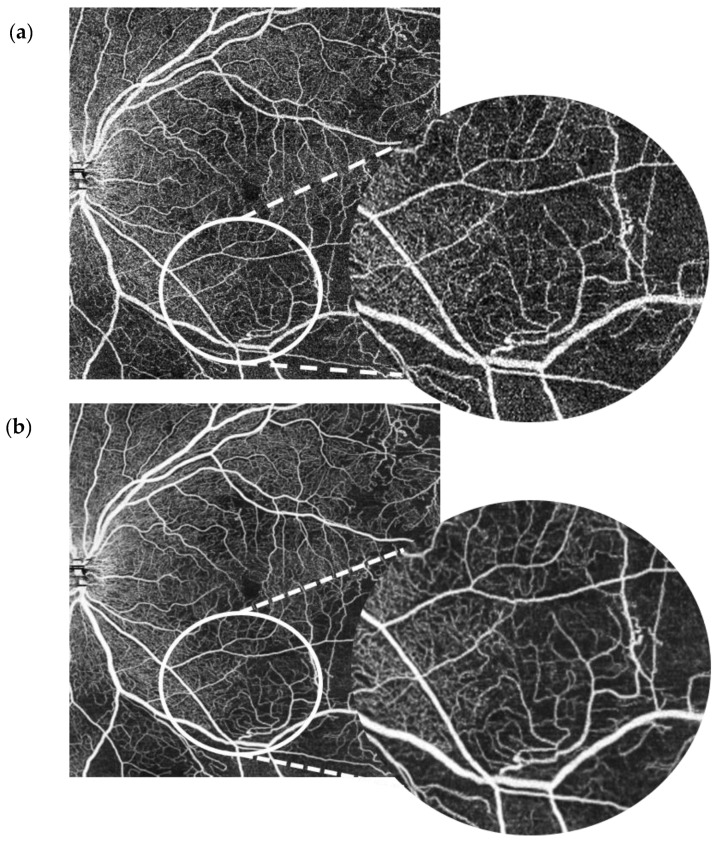
10 × 10 mm OCT-A scan of the superficial capillary plexus in a patient with systemic lupus erythematosus retinopathy. Baseline image (**a**) vs. denoised image (**b**).

**Figure 2:**
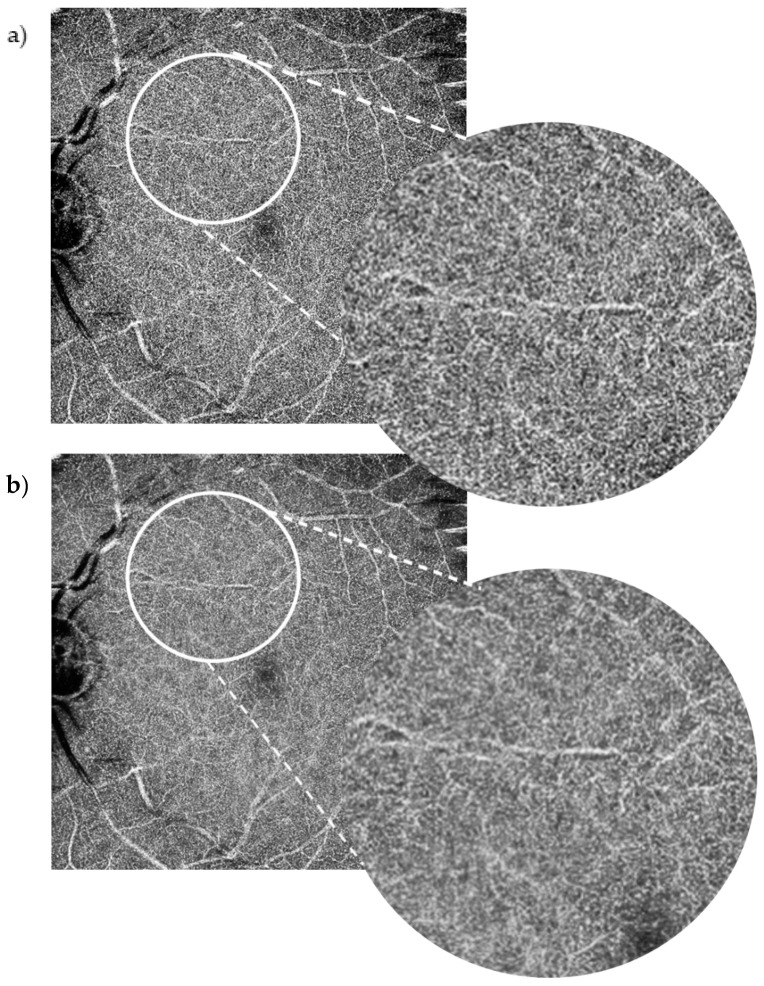
10 × 10 mm OCT-A scan of the deep capillary plexus. Baseline image (**a**) vs. denoised image (**b**). The positive impact of the denoise software on the Superficial Capillary Plexus (SCP) did not apply to the same extent to the Deep Capillary Plexus (DCP). Although the more clinically standard smaller field DCP scans were of higher quality, the widefield 10 × 10 mm images for this study showed shadowing from the SCP and relative loss of clarity.

Each patient had wide-field (10 × 10 mm) macula OCT-A scans on the Canon Xephilio OCT-A1 machine with 10-layer automated segmentation and a refresh rate of 70,000 A-scans/second. Depth of field of view was set to 10 × 10 mm with an axial sampling density of 464 × 464 pixels. The number of repetitions was set at 2. The position of the field investigated was centred on the foveal region. En face OCT-As were segmented automatically using built-in software that defines each different retinal layer. The enhanced artificial image was automatically created by selecting the “denoise” button after image acquisition to initiate the algorithm, which completed processing in approximately 2 s. The same technician captured the scans of each patient in this way. All OCT-A images were qualitatively examined for severe motion or shadow artefacts. Figure 1 and Figure 2 demonstrate denoised images created this way along with their corresponding baseline images.

### 2.3. Network Architecture of Deep Learning Denoising Method and Training Protocol

This study uses the same algorithms for denoise that Kadomoto et al. [14] used in their study, which compared the Superficial Capillary Plexus (SCP) between original, averaged, and denoised 3 × 3 mm OCT-A scans. CNN architecture was employed to intelligent denoise. This is an encoder-decoder style neural network that undertakes semantic segmentation. First, an encoder took an image tile as input and successively computed feature maps on multiple scales. Second, a decoder took the feature representation and classified all pixels/voxels at the original image resolution in parallel. The layers in the decoder synthesized the image, starting at low-resolution feature maps and moving to full-resolution feature maps. In short, it is comparable to a regression analysis where the trained CNN of the Canon OCT-A1 uses algorithms to automatically create images similar or even superior in quality to the inputted averaged images [14]. For training, sets of standard OCT-A images as well as averaged OCT-A images were input. A total of 23,744 data points were selected from a set of 742 patients (diseased: 595, healthy: 147). The diseased OCT-A image data was obtained at Kyoto University Hospital, Japan and Tokyo Women’s Medical University Hospital, Japan. Training was performed using a computer with 64 GB of RAM, 4TB HDD, and an NVIDIA 1080Ti 11GB Graphics Processing Unit. The Intelligent Denoise software converted the noisy input en face OCT-A images into denoised images. The Canon OCT-A1 has the same specification in hardware and software as the OCT-HS100 used in Kadomoto’s study, and the same denoise algorithms were utilized to the OCT-A1.

### 2.4. Expert Image Assessment

For subjective OCT-A image quality assessments, a relative or comparative evaluation is known to be easier and more reliable than an absolute evaluation or scoring of image quality [16]. For this reason, we used a paired comparison approach to assess OCT-A image quality. Two medical retinal experts (TA and AG) masked to the image information performed comparisons of original and denoised image pairs. The medical retina experts graded 49 pairs of en face OCT-A images in total. They were presented by a clinical research fellow (OM, WS) on a standard monitor. The images were presented in two panels (left and right) to facilitate comparison with random assignment of the original versus denoised images to the left and right panels. The assessment was stratified to the Superficial Capillary Plexus (SCP) and Deep Capillary Plexus (DCP). The SCP layer was further divided into categories of 1st- and 2nd-order vessels, retinal capillaries, and, finally, any areas of specific pathology. The DCP layer was divided into retinal capillaries and any areas of specific pathology. Each of these categories was analyzed one quadrant at a time, and for each category, the original image was compared with the same image with the intelligent denoise software applied. For each image pair and for each layer and category, the retinal experts were asked to make a clinical judgement of the following parameters: (1) any true clinically meaningful anatomical features from the original image that were lost on the enhanced image, (2) any false clinically meaningful artefactual anatomical features created in the enhanced image, (3) any perceived overall preferred image for clinical assessment, (4) any further comments or observations. A comparative image quality score was assigned to each image pair as follows: 1 = the left image is better, 0 = the two images are equal; −1 = the right image is better. Both SCP layers and DCP layers were assessed in this manner. If there was disagreement in independent decisions, graders debated and compromised to agree on a single determination. There was no instance where this was not possible. When the DCP layer was analyzed, a Canon-OCT-A built-in projection artefact removal feature was applied. A proforma (see Appendix A) was used to record the above data for each patient’s scan.

## 3. Results

One consecutive OCT-A scan had a Canon imaging score of 4 out of 10. This did not meet the inclusion criteria in image quality (Canon image quality rating of above 4/10), so it was excluded, leaving 49 scans from one eye of each of the remaining 49 patients for further analyses. The average Canon image quality was 7. The standard deviation of the image quality was 1.15.

**Figure 3:**
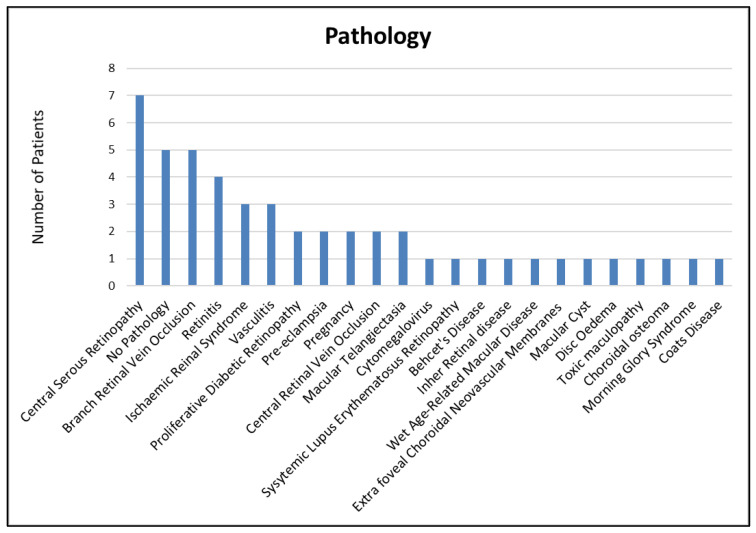
Pathology assessed in forty-nine 10 × 10 mm OCT-A1 scans.

The patients had a mean age of 48 years (range: 15–87 years). Twenty-three were male and 27 were female. Thirty-nine patients were Caucasian, 6 were black, and 5 were Asian. Thirty-two scans (65%) had evident signs of retinal pathology, with 17 scans (35%) found to have no pathology. The various pathologies present in the 49 scans are summarised in Figure 3.

**Figure A2:**
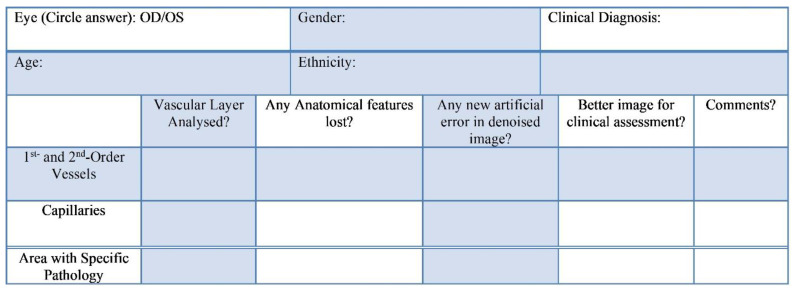
Proforma used by the retinal experts when comparing baseline to denoised OCT-A images.

The SCP layer was analysed before and after denoising in 49 scans. Comparison between the original and the denoised image was documented using a proforma as seen in the Appendix A, Figure A2. The denoised DCP of the same 49 scans was similarly analysed and compared with the original image. Please see Figure 2 for an example of an original OCT-A scan of the SCP compared with its denoised equivalent. Please see Figure 3 for an example of an original OCT-A scan of the DCP compared with its denoised equivalent.

In reporting our primary endpoints, we found, according to clinical acumen, that no clinically meaningful anatomical features were lost, and no new false features were found in the enhanced image compared with the original, in any category. This was the case with the 1st- and 2nd-order vessels, capillaries, and any area of specific pathology present in either the SCP or the DCP. There was no difference between quadrants when each quadrant of the original image was compared with its denoised equivalent. When selecting the best image for clinical assessment, all evaluations recorded either preference for the enhanced image or else no preference for either scan. Never was the original image selected as superior to the denoised image by the retinal experts. For SCP 1st- and 2nd-order vessels, preference for the enhanced image was 33%. There was no difference seen between images in the remaining 67%. For the SCP capillaries, preference for the enhanced image was 98%. No difference was seen in the remaining 2%. For the SCP areas of specific pathology, preference for the enhanced image was 75%. There was no difference found between images in the remaining 25%. For the DCP capillaries, preference for the enhanced images was 35%. There was no difference seen between images in the remaining 65%. For the DCP areas of specific pathology, preference for the enhanced image was 28%. No difference was seen in the remaining 72% of images. The study results are summarized in Table 1.

## 4. Discussion

OCT-A technologies show great potential for clinical use in the diagnosis and management of retinal disease, and optimisation of images could further enhance their utility. Previous studies examining methods that attempt to reduce background noise and improve OCT-A contrast quality have shown benefits of “multiple imaging averaging” techniques [13,17]. However, acquiring these multiple images is labor- and time-intensive, which is itself not conducive to the highest quality images [18] and can be burdensome on both the patient and technician [10], particularly in a busy clinical environment.

The use of artificial intelligence algorithms to enhance images could provide faster, more practical means of image enhancement. In this study, we compared 10 × 10 mm OCT-A scans of retinal vasculature with processed images that had intelligent denoise software algorithms deployed. We looked at the effect of denoise on the SCP as well as the DCP and its effect on different forms of vasculature. In our study, the intelligent denoise feature of the Canon Xephilio OCT-A1 appears to allow for a rapid facility to improve image quality with no evidence of meaningful loss of clinical detail nor new artefactual errors. This is particularly evident for the superficial plexus and for the finer capillaries rather than larger vessels.

Our study, which analysed 10 × 10 mm scans, is the first to analyse the effect of denoise on original OCT-A images exceeding 3 × 3 mm in a clinical setting. Improving the image quality of OCT-A scans with wider fundal views has the potential to greatly benefit clinical care [19]. OCT-A can assess peripheral fundal changes due to diabetic retinopathy, or other causes of retinal ischemia [9] and peripheral retinal neovascularisation. Furthermore, our study uniquely evaluated imaging of the DCP as well as the SCP. OCT-A has identified the significance of microvascular changes in the DCP in several retinal vascular diseases based on the depth-resolved imaging technique [20,21]. We found that overall, the positive impact of the denoise software on the SCP did not apply to the same extent to the DCP; in most DCP image comparisons (65%), there was no improvement noted between the DCP denoised images and DCP original images. We hypothesise that this may be due to shadowing from the superficial plexus and the large 10 × 10 mm field of view we used in this study, which may have resulted in poorer DCP resolution. The Canon Xephilio OCT-A1 is undergoing developments to improve its wide-field DCP imaging quality.

Our study has limitations that should be considered when assessing the findings. The sample size of patients was small (49 patients), but it did consist of a wide range of pathologies. The clarity of the DCP was not as distinct as the SCP. Improvements to OCT-A devices are necessary in parallel with improvements in deep learning technology.

Despite these limitations, our study provides evidence that, in comparison to clinically analysing only original OCT-A images, deep learning denoise algorithms may enhance the original 10 × 10 mm OCT-A scans to aid the clinician in their diagnosis and treatment of retinal pathology. We demonstrate the ability of a deep learning model to efficiently improve the original OCT-A image quality with no clinically meaningful artefactual aberrations complicating clinical assessment. Overall, this study provides evidence to suggest that, especially for superficial plexus, denoising algorithms are an effective means of improving the clinical quality of the original, single-shot OCT-A image.

## Figures and Tables

**Table 1:** Perceived better image overall for clinical assessment.

Vascular Layer And Vessel Category Analysed	Image Selected as Best for Clinical Assessment (%)		
**Superficial Capillary Plexus**	Denoised Image	Original Image	No difference
1st-and 2nd-Order Vessels (49)	33% (16)	0% (0)	67% (33)
Capillaries (49)	98% (48)	0% (0)	2% (1)
Area with specific pathology (32)	75% (24)	0% (0)	25% (8)
**Deep Capillary Plexus**	Denoised Image	Original Image	No difference
Capillaries (49)	35% (17)	0% (0)	65% (32)
Area with specific pathology (32)	28% (9)	0% (0)	72% (23)

**Figure A1:**
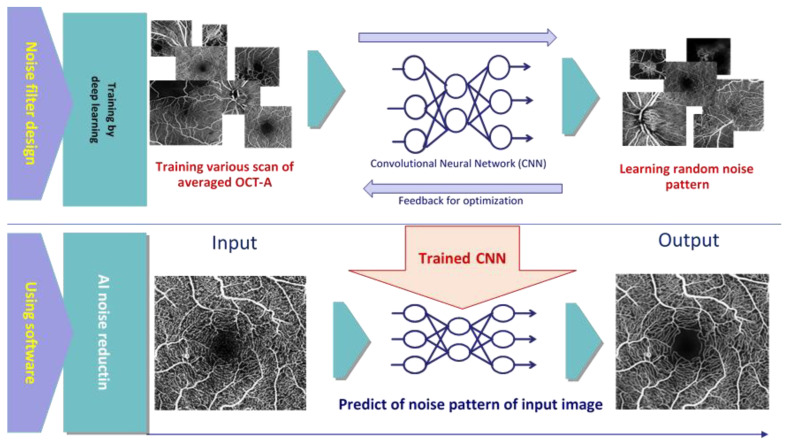
Deep learning denoise model schematic.

## Data Availability

Data available on request due to ethical restrictions. The data presented in this study are available on request from the corresponding author. The data are not publicly available due to ethical restrictions.
